# Ultra-Rapid Vision in Birds

**DOI:** 10.1371/journal.pone.0151099

**Published:** 2016-03-18

**Authors:** Jannika E. Boström, Marina Dimitrova, Cindy Canton, Olle Håstad, Anna Qvarnström, Anders Ödeen

**Affiliations:** 1 Department of Animal Ecology, Uppsala University, Norbyvägen 18D, S-752 36, Uppsala, Sweden; 2 Department of Zoology, Stockholm University, S-106 91, Stockholm, Sweden; 3 Department of Anatomy, Physiology and Biochemistry, Swedish University of Agricultural Sciences, Box 7011, S-750 07, Uppsala, Sweden; University Zürich, SWITZERLAND

## Abstract

Flying animals need to accurately detect, identify and track fast-moving objects and these behavioral requirements are likely to strongly select for abilities to resolve visual detail in time. However, evidence of highly elevated temporal acuity relative to non-flying animals has so far been confined to insects while it has been missing in birds. With behavioral experiments on three wild passerine species, blue tits, collared and pied flycatchers, we demonstrate temporal acuities of vision far exceeding predictions based on the sizes and metabolic rates of these birds. This implies a history of strong natural selection on temporal resolution. These birds can resolve alternating light-dark cycles at up to 145 Hz (average: 129, 127 and 137, respectively), which is ca. 50 Hz over the highest frequency shown in any other vertebrate. We argue that rapid vision should confer a selective advantage in many bird species that are ecologically similar to the three species examined in our study. Thus, rapid vision may be a more typical avian trait than the famously sharp vision found in birds of prey.

## Results and Discussion

**Fig 1:**
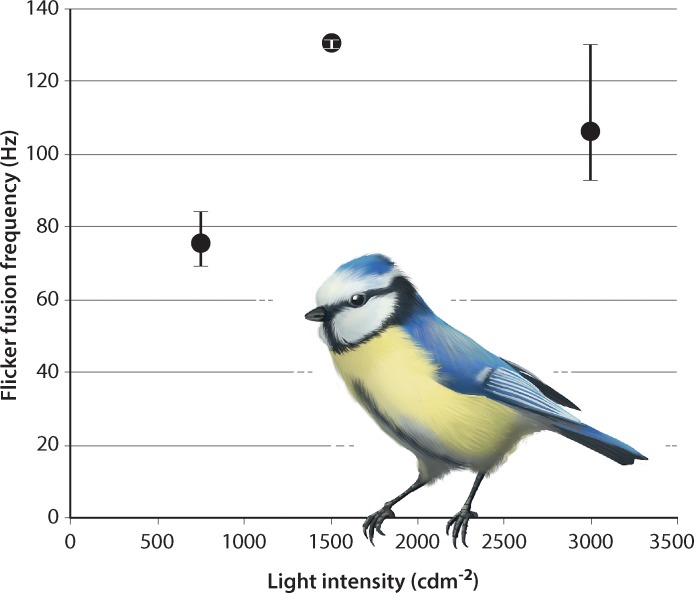
Flicker fusion frequencies for blue tits by three different light stimuli intensities. Averages and ranges are shown with filled circles and brackets, respectively. Twelve individual blue tits were tested once at one of the light intensities 750, 1500 (n = 3) and 3000 cdm^-2^ (n = 6). The critical flicker fusion frequency (CFF), with a maximum of 131 Hz and 130.3 ± 0.94 Hz (±SD) on average, was reached at 1500 cdm^-2^.

**Fig 2:**
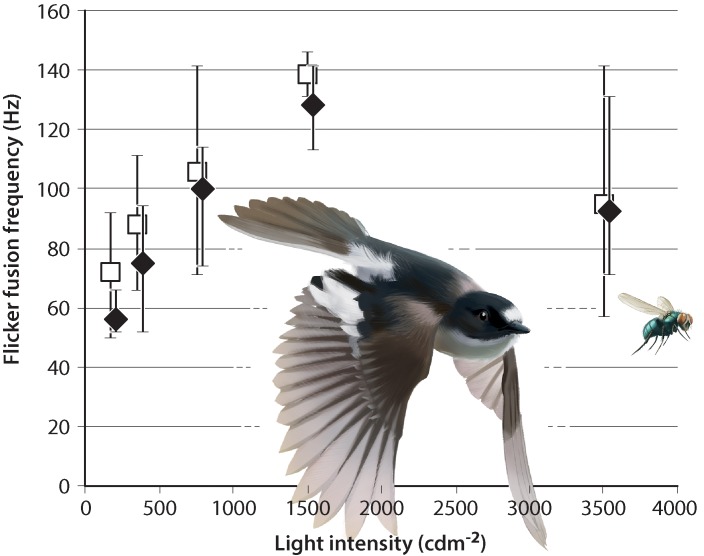
Flicker fusion frequencies for collared (closed diamonds) and pied flycatchers (open squares). Averages are shown together with ranges (brackets). Seven collared and eight pied flycatchers were repeatedly tested at up to five different light intensities each: 160 (n = 5 collared + 4 pied), 350 (n = 7 + 4), 750, 1500 (n = 7 + 8) and 3500 cdm^-2^ (n = 6 + 7). The critical flicker fusion frequency (CFF), with a maximum of 141 Hz and 128.1 ± 9.8 Hz (±SD) on average for the collared flycatchers and up to 146 Hz and 138.2 ± 6.5 Hz on average for the pied flycatchers, was attained in both species at 1500 cdm^-2^.

We have performed behavioral experiments to estimate temporal resolution of the complete visual pathway in three species of small passerine birds: blue tit (*Cyanistes caeruleus*), collared flycatcher (*Ficedula albicollis*) and pied flycatcher (*F*. *hypoleuca*). We used an operant conditioning approach in which the birds were trained and tested for the task of distinguishing flickering from constant stimuli produced by LED-arrays simulating daylight. Flickering and constant lamps became indistinguishable at frequencies of up to 131 Hz for blue tits (Fig 1), 141 Hz for collared flycatchers and 146 Hz for pied flycatchers (Fig 2). All three species had the highest flicker fusion frequencies (the critical flicker fusion frequency: CFF) at the same light intensity, 1500 cdm^-2^. The average CFFs of these species, 130.3 ± 0.94 Hz (±SD) in three blue tits, 128.1 ± 9.8 Hz in seven collared flycatchers and 138.2 ± 6.5 Hz in eight pied flycatchers, are clearly higher than those of humans (around 50–100 Hz depending on stimulus size [1]) and around 40 Hz higher than for any other vertebrate tested to date [2, 3].

High CFF has been shown to correlate with high metabolic rate and small body size, predicting that vertebrates with the size and metabolic rate of small passerines should perceive visual flicker up to 100 Hz [2]. Our results however exceeded the CFFs that one would predict, given the sizes and metabolic rates of the species tested, by 30–50% (95–97 Hz) [2], implying an evolutionary history of strong selection for maximizing temporal acuity.

All three species are agile flyers, active during daylight and regularly navigate at high speed through dense forest. Blue tits are insectivorous during the breeding season and flycatchers throughout the year, regularly catching prey on the wing. High temporal resolution of the visual system is necessary for fast-flying and maneuvering organisms, which need to rapidly integrate information [4] to allow accurate pattern recognition [5], motion tracking [2] and depth perception, which in birds is maintained through optic flow [6]. Extremely high temporal acuity of vision has previously been demonstrated in some insects [7]. However, many birds should also be under selection for this trait, not least those catching fast-flying insects on the wing and therefore attempting to match the insects’ aerobatics, or having to avoid motion blur when navigating through dense vegetation or other complex environments. The daylight activity typical of most bird species is reflected by retinas rich in cones, which have more than four times faster post-stimulus recovery rates than rods [8]. Birds’ fast metabolism and small sizes both increase maneuverability, and their high metabolic rates also enable fast changes in the photoreceptor membrane [2].

Although the airborne and diurnal lifestyles of birds have long been suggested to favor high temporal acuity of vision [9–11], empirical evidence for generally higher CFFs than in other vertebrates has been lacking. Only Dodt’s and Wirth’s [12] electroretinograms (ERGs) reaching about 140 Hz in the pigeon, *Columba livia*, exceed the range of CFFs recorded in other vertebrates (see [2, 13]), but may not be directly comparable to behavioral results. In vertebrates, ERGs produce accurate estimates of retinal response but exclude temporal summation of signals from the retinal neurons leading to results significantly higher than those based on behavioral assays [14]. One reason for the lack of evidence of extreme temporal acuity in birds may be that few studies have reached CFF by using sufficiently high light intensities for temporal acuity to peak [15]. Another reason may be that behavioral studies have focused on species that forage on immobile or slowly moving food items or live in low-light conditions: (average highest frequency measured) budgerigar (69 Hz) [16], pigeon (75 Hz (from graph)) [17] and chicken (90 Hz [18] (from graph, white light) and 87 Hz [15]).

**Fig 3:**
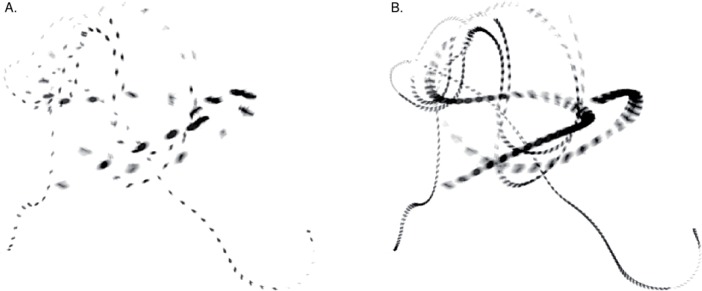
The flight paths of two blue bottle flies (*Calliphora vomitoria*) sampled from high-speed video (S1 Movie): A) at the rate of the visual system of a human (40 frames/s) and B) at the rate of a pied flycatcher (120 frames/s) at a light intensity of approximately 500 cdm^-2^. The flycatcher refreshes visual input almost three times faster, resulting in a much more detailed view of the flight paths of the flies.

The behavioral requirements of birds in flight to accurately detect, identify and track objects whose image moves rapidly over the retina are likely to select for abilities to resolve visual detail in time rather than space. Visual performance is ultimately limited by the ambient light level. In addition energy constraints present a limiting factor (c.f. [19]), possibly exacerbated in birds by their large and avascular retinas. This should result in trade-offs between different aspects of vision that explain why birds in general, compared to raptors, have quite poor spatial resolution [20]. Eagles, which forage in open and brightly lit habitats, have developed the sharpest vision known [20, 21], allowing them to spot prey over long distances. However, tracking objects moving at high angular velocity should take priority when hawking for insects and while flying in complex environments, for instance when small passerines head for cover in dense vegetation. Motion tracking is improved by increasing visual refresh rates (Fig 3), but since this action decreases the visual integration time it becomes necessary to integrate over larger receptive fields to maintain the photon catch, leading to lower spatial acuity [22]. This is particularly true as light levels fall. We therefore expect fast-eyed species like tits and flycatchers to have compromised spatial acuity, the lower limit of which should be determined by the need to identify behaviorally important cues, such as prey or twigs and other obstacles in the flight path.

To the best of our knowledge, the temporal resolution data presented here are the first available for actively flying birds that feed on fast-moving prey. Our study is also the first to determine the CFF of the vision system of wild birds with predominantly diurnal habits. Thus, many other ecologically similar bird species may have been driven to such physiological extremes, either by flying in complex environments or due to an evolutionary arms race between predatory species and their prey. The fast vision of these small passerines may very well be a more typical avian trait than the sharp spatial vision found in birds of prey.

## Methods

### Animals

#### Blue tits

The blue tits were captured with mist nets at Tovetorp Research Station (Stockholm University) in South-Eastern Sweden (58°56’N, 17°08’E) during November 2010 –March 2011 and during October 2011 –March 2012. The birds were kept individually in indoor cages (40×60×80 cm), the room temperature was about 18°C and the light:dark rhythm (with 30 minutes dawn and dusk) was adjusted according to the prevailing day length using high frequency (>20 kHz) fluorescent lighting. The home cages were equipped with three perches and the birds had access to suet, sunflower seeds, peanuts and water *ad libitum*. The birds were kept in captivity for a maximum of 14 days, before they were ringed and released in the area of capture.

#### Flycatchers

Flycatchers were caught on the island of Öland in the Baltic Sea (58°10’N, 16°58’E) during April and May 2013, and May and June 2014, either with traps in nest boxes or with mist nets. All individuals were ringed and measured following a protocol used for an on-going survey of the populations [23]. The birds were kept indoors, individually in cages (45×65×75 cm), and were given mealworms and water *ad libitum*. Each cage had one perch located 49 cm from the light stimuli and 17 cm from the cage floor. Ambient light was provided by high frequency fluorescent lighting, which was turned on at 6 a.m. and turned off at 9 p.m., with a one-hour period of dawn and dusk. All individuals were kept and tested for a maximum of 14 days before they were released back into the wild.

### Determining flicker fusion frequency by operant conditioning

Light stimuli were 5 mm white (Avago technologies, Malaysia) and UV (Roithner LaserTechnik GmbH, Vienna, Austria) LED’s attached to the distal end of 18 mm diameter aluminum tubes, with a UV-transparent Perspex panel at the proximal end. LED’s were combined in groups of up to six to match natural daylight as perceived by the birds (S1 Fig). Illuminations were altered using neutral density (25, 50 and 75%) and diffusion filters (Lee Filters, Andover, UK) and confirmed using a spectroradiometer (AvaSpec-2048 connected to an Avantes CC-UV/VIS cosine corrector; AvaSoft 7.0 computer software), as well as through altering the length of the aluminium tubes (40–120 mm). The light stimuli were controlled by function generators (2 MHz, GW Instek, Suzhou, China and 3 MHz, TENMA, Taiwan) and light intensities in the cages were measured using a Hagner ScreenMaster instrument (B. Hagner AB, Solna, Sweden). The ambient light intensities in the cages were kept at maximum 10% of the stimulus illumination.

#### Blue tits

The birds were held individually in separate holding cages between experiment sessions. Training and testing were performed individually in a Skinner box measuring 55×70×90 cm, with *ad libitum* access to water. The birds were food deprived for 1 h prior to each session to increase their motivation to perform the task. The light stimuli were situated 15 cm apart at floor level on a short side of the cage. Below each light panel the food reward, a peanut piece, was placed in a hole in a piece of cardboard. During training the hole was covered with a piece of 50% black paper measuring 1.5×1.5 cm. The training was conducted in four steps, with the peanut placed: 1) visible on top of the paper, 2) visible under the paper but not inside the cardboard, 3) visible inside the hole in the cardboard, and 4) fully covered. The blue tits were exposed to two light stimuli–one perceived as constant (flickering at more than 2 kHz) and the other flickering at 50 Hz–and trained with positive reinforcement to prefer the constant stimulus. The constant stimulus was used since the birds displayed such a strong initial reluctance to approach the flickering stimulus that training for it was impossible within the permitted timeframe. In both training and testing the positive stimulus was randomly assigned to either the right or left LED-array. When the birds managed to choose the correct stimulus four times out of five in the first setup, they were moved to the next training step. This procedure was repeated until the birds had completed all four training steps and were ready for the experiment.

Testing started at 50 Hz and the frequency was then raised to 80 Hz, 100 Hz and 120 Hz as long as the bird chose the correct light stimulus by lifting the cover over the reward four times out of five. When the bird failed to do so, the frequency was decreased to the last value where the bird was successful and then raised again in steps of 10 Hz at a time, 5 Hz at a time and in the end 1 Hz at a time until the threshold value was determined. Each individual was trained and tested at a single light intensity, either 750, 1500 or 3000 cdm^-2^ (S1 Table).

#### Flycatchers

The birds were held individually and both training and testing was conducted in the holding cages. They were food deprived for 1 h prior to training and tests to increase their motivation to perform the task. During training the birds were presented two light stimuli, one perceived as constant and one flickering at 40 Hz, and trained to approach either the constant or the flickering stimulus by positive reinforcement using mealworms as a food reward in a remote controlled feeder below each light stimulus. All the flycatchers were trained and tested at up to five light intensities: 160, 350, 750, 1500 and 3500 cdm^-2^ (S1 Table) in random order. The procedure was the same as for blue tits and the frequency of the flickering light was increased in steps of 20 Hz, decreased upon failure and increased again in steps of 5 Hz and 1 Hz until the flicker fusion frequency (FFF) for that particular light intensity was reached.

The experiments described in this study, including handling in the field, were specifically approved by the regional animal ethics committee, Linköpings djurförsöksetiska nämnd, with permissions numbers 53–10 and 152–10 (regarding blue tits) and 10–13 (regarding pied and collared flycatchers). The birds were captured with permission from the Swedish Museum of Natural History (Dnr 52-00060/2010, ringing license no. 605) and the Swedish Environmental Protection Agency (NV-02858-14).

### Predicting flicker fusion frequency from physiology

Healy *et al*. [2] recently demonstrated a correlation in vertebrates between critical flicker fusion frequency (CFF) and body weight, basal metabolism and light intensity, without a significant contribution from phylogeny. From a generalized linear model based on Healy *et al*. [2] and body weights and metabolic rates from the literature, we predicted CFF for our three species. A body weight of 15 g and a metabolic rate of 20.7 kJ/day [24], giving a weight-adjusted basal metabolic rate of 0.016 W/g, predicted a CFF of 95 Hz for the pied flycatcher, 14 g and 23.9 kJ/day [25], giving 0.021 W/g and 96 Hz for the collared flycatcher, and 11 g and 20 kJ/day [26], giving 0.020 W/g and 97 Hz for the blue tit.

## Supporting Information

S1 Fig Spectrogram of the LED-based light stimulus used in this study. (PDF)

S1 Movie A high-speed video clip of two blue bottle flies (*Calliphora vomitoria*), a male and a female, chasing each other, first at “normal” speed as perceived by humans and then at a reduced speed to visualize how they might appear to a flycatcher with its higher visual refresh rate. A segment from this video is illustrated in Fig 3.
(MP4)

S1 Table Flicker fusion frequency (FFF) at each tested light intensity for each individual bird (ring number). (CSV)
