# Supplementary figures and images for: Ultra-Rapid Vision in Birds
Source: PLoS One. 2016 Mar 18;11(3):e0151099. doi: 10.1371/journal.pone.0151099 (PMC4798572; doi:10.1371/journal.pone.0151099)

Normalized quantum emission

1.00  
0.75  
0.50  
0.25  
0.00

300

400

500

600

700

Wavelength

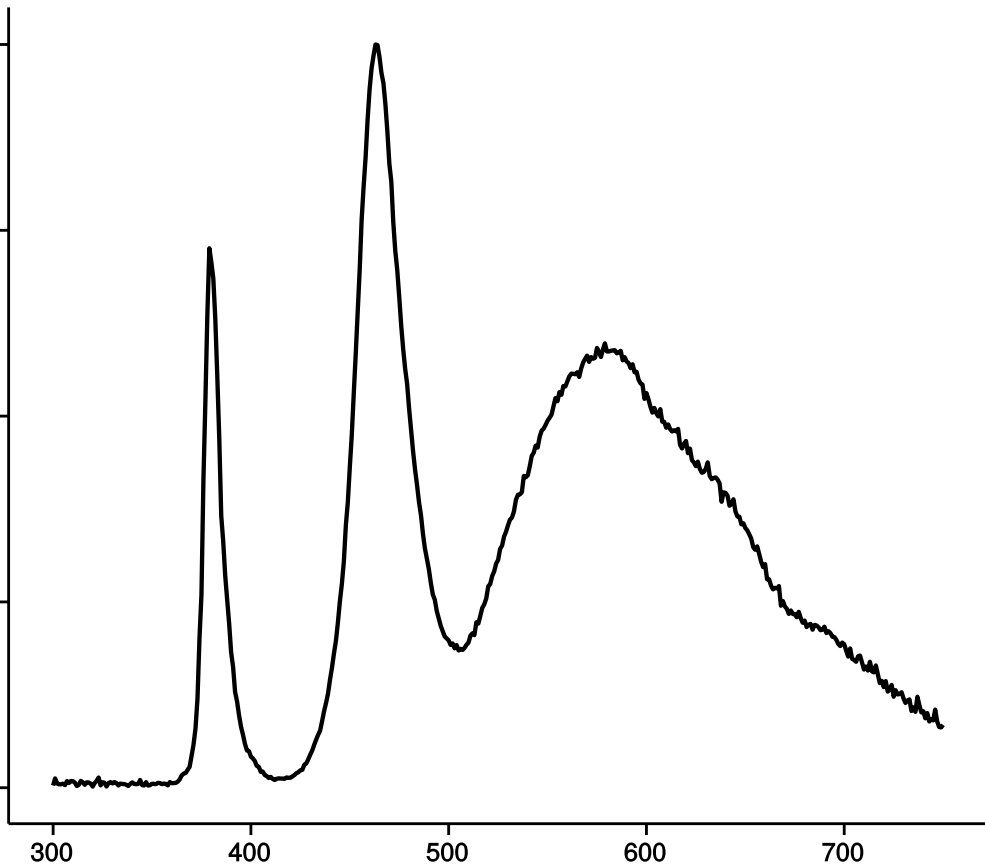

Supplement: S1 Fig — (PDF) [file pone.0151099.s001.pdf]
